# COVID-19 pandemic and medical education: A medical student’s perspective

**DOI:** 10.36834/cmej.70242

**Published:** 2020-09-23

**Authors:** Jad Abi-Rafeh, Tyler Safran, Alain J. Azzi

**Affiliations:** 1 Faculty of Medicine, McGill University, Quebec, Canada; 2 Department of Surgery, Division of Plastic and Reconstructive Surgery, McGill University, Quebec, Canada

## Abstract

The specific impact of the COVID-19 pandemic on medical education remains elusive and evolving. Clinical teaching opportunities have become limited with the shift in focus of supervising physicians away from trainees and towards the care of the sick and vulnerable. The presence of medical students in hospitals has come to represent an added strain on vital resources, and the added risk of viral dissemination into communities has left medical students eager to help observing from only the sidelines. The present article provides a medical student’s perspective on this unique, evolving situation, and identifies several learning opportunities that medical students may reflect upon and carry forth into their careers ahead. By exploring the current and future impact of this pandemic on clerkship, pre-clerkship and post-graduate medical training, specific challenges and future direction for both medical students and educators are discussed.

## Abstract (fr)

L’impact précis de la pandémie de la COVID-19 sur la formation en médecine reste insaisissable et en constante évolution. Les opportunités d’enseignement clinique sont devenues limitées étant donné le changement de priorité demandé aux médecins enseignants, les éloignant de la supervision des étudiants pour se concentrer aux soins des malades et des personnes vulnérables. La présence d’étudiants en médecine dans les hôpitaux représente une pression accrue sur les ressources essentielles et ajoute un risque de propagation du virus dans les collectivités. Par conséquent, les étudiants en médecine qui sont prêts à apporter leur aide ne peuvent qu’observer de loin. Le présent article explique la perspective d’un étudiant en médecine sur cette situation sans précédent et toujours en évolution et identifie les plusieurs opportunités d’apprentissage auxquelles les étudiants en médecine peuvent réfléchir et retenir pour leur future carrière. Il traite également de l’impact actuel et futur de cette pandémie sur l’externat, le préexternat et la formation médicale post-doctorale, de défis particuliers et desorientations futures pour les étudiants en médecine et des éducateurs.

The COVID-19 pandemic has taken the world and medical community by storm. In December 2019, SARS-Cov-2, a coronavirus strain believed to originate from bats, crossed over to humans at a wet animal market in Wuhan, China, and began spreading within the region and beyond.^1^ By March 11^th^, 2020, the World Health Organization declared COVID-19 a pandemic.^2^ With millions of cases recorded and hundreds of thousands of deaths, the world has now found itself at odds with a virus more formidable than anything encountered in the past centruy.^3^^,^^4^

At first, in North America, the medical community observed at a distance the shocking despair reported from the pacific, empathized with the efforts and sacrifices of our healthcare colleagues in China, and was astonished by the speed at which dedicated treatment facilities emerged. Containment efforts were strict, and in some instances harsh, but the world observed calmly with what appeared to be a sense of reassurance; “it won’t be that bad here,” many of us overheard in our busy hospital halls. Slowly, however, cases began to surface at home, though limited and isolated at first; we remained calm. A sudden surge was then reported in Europe, and a few days later, we heard accounts of doctors in Italy having to choose who and who not to save, just like war. Cases at home continued to rise. Was it now time to get concerned? Governments began closing borders and limiting travel. Social distancing advisories were enacted, and recommendations slowly became law. Every non-essential worker was now to stay at home: having failed prudent testing, identification and containment efforts, social distancing was now our only hope to give the vulnerable in our communities a fighting chance.

With every development reported, my medical student colleagues and I grew more motivated to stand alongside our great physicians, nurses, and other healthcare partners in confronting this pandemic. However, eventually, we received a communication many of us were dreading: medical students were to be withdrawn from all clinical duties.

Although disappointing, this decision did not come as a surprise. While our usefulness in hospitals has grown as we’ve progressed through our medical training, our fundamental role as learners cannot be ignored. Education opportunities became limited with the cancellation of clinics and operating room cases; the priority of physicians was now the care of the sick and vulnerable, rather than the supervision of medical students. For any form of patient care we would help deliver, direct physician oversight was necessary, thus representing an added strain on hospital resources and protective equipment already being depleted. Finally, the presence of learners in hospitals can represent an added, unnecessary risk for viral dissemination into our communities. We thus found ourselves admiring the arduous work of our supervisors as usual, but now, from the confinement of our homes, faced with the chilling reality that no matter our motivation and desire to help, our biggest contribution to society was to keep away from the hospital. This was a difficult pill for many of us to swallow, given that our choice of career is driven by our desire to be at the service of others. Nonetheless, and in accepting the present circumstances, we must reflect upon this unique, unfortunate situation, and identify learning opportunities we may carry forth with us into our careers ahead.

Firstly, this pandemic is a stark reminder of how the fundamental role of every physician transcends subspecialty: to heal and serve those most vulnerable. Both surgeons and internists are now seen working in intensive care units and emergency departments. Focused on how to best prepare for our residency applications, we medical students often find ourselves losing sight of our primary goal of becoming astute, well-rounded physicians first. We must embrace every clinical rotation while it lasts, and every teaching opportunity as a privilege. We are reminded of the sacrifices physicians continue to make daily, and in witnessing the impact of their virtuous work, can come to appreciate the true meaning of medical practice.

In considering the impact of this pandemic on medical education, much remains elusive. Fourth-year medical students on track to beginning their residency training shortly may find themselves offered the opportunity to graduate early and join the hospital workforce; serving on COVID-19 wards can integrate well into training requirements of many first-year residents. In contrast, the impact on third-year medical students may be more significant and difficult to manage, given that simulation and virtual teaching of clinical skills on a large scale remains a substantial challenge.^5^ As faculties struggle to avoid delaying graduation in making up for clinical time away from core clerkship rotations, third-year medical students may find themselves losing a significant amount of fourth year electives in order to meet degree requirements. Indeed, a nation-wide moratorium on fourth-year visiting electives has been adopted in Canada, representing a potential challenge for post-graduate programs seeking to recruit applicants this upcoming match cycle. For first- and second-year medical students for whom a rapid transition towards online instruction has been completed, the impact on pre-clinical training may be minimized by swift leveraging of existing technological capabilities and video conferencing services, as recently demonstrated.^5^ Several studies have reported on the utility of online educational resources, instructional videos services, teaching platforms, and smart phone / tablet applications that supplement medical training curricula.^6^^-^^8^ With the advent of social media and growing online presence of physicians, educators, and trainees, medical students may remain connected to clinical learning albeit through a computer or smartphone screen.

The duration of this situation remains uncertain as the pandemic continues to unfold. Forced to observe from only the sidelines, medical students eager to help are awaiting impatiently the opportunity to again be at the service of others. However, and although at a distance from patients, medical students may continue cultivating their work ethic and compassion by addressing evolving needs of our communities. Indeed, several student-led initiatives continue to emerge, from food delivery services for hospital staff hard at work, to peer-peer teaching and meals-on-wheels programs to ensure food security among the elderly and those most vulnerable. Armed with the most recent evidence, we can help spread awareness to reinforce the public health strategies necessary to contain this pandemic, and, with some training, provide non-clinical support such as contact tracing. Despite observing from only the sidelines as we impatiently await our return to clinical care, we can still play our part, for, as Charles Dickens once wrote, “no one is useless in this world who lightens the burdens of another.”
